# MRC-Net: a reliable plant disease classification framework with multi-frequency state-space enhancement and conformal prediction

**DOI:** 10.3389/fpls.2026.1895287

**Published:** 2026-09-03

**Authors:** Shiyao Xie, Xiaohong Zhang, Yang Li

**Affiliations:** 1 School of Hydraulic and Electric-Power, Heilongjiang University, Harbin, China; 2 North-Eastern Federal University, Yakutsk, Russia; 3 Heilongjiang Province Hydraulic Research Institute, Harbin, China

**Keywords:** conformal prediction, multi-frequency feature enhancement, plant disease classification, reliable prediction, state-space model

## Abstract

Plant disease image classification is a key task in disease monitoring and precise prevention and control in smart agriculture. However, complex backgrounds, finegrained lesion differences, and model overconfidence still limit recognition performance and practical application reliability. To address these issues, this paper proposes a reliable plant disease recognition framework that integrates multi-frequency selective state-space feature enhancement with conformal-aware reliable prediction. The proposed method adopts Swin-Tiny as the backbone network and uses the MF-SSFE module to jointly model low-frequency leaf structures, high-frequency lesion textures, and cross-region state-space contexts, thereby enhancing disease-related evidence in complex scenarios. Meanwhile, the CARP module is introduced to incorporate the idea of conformal prediction into the classification output process, enabling the model to express uncertainty while providing class predictions. Experimental results show that the proposed method achieves Accuracy values of 0.9891, 0.3889, and 0.9800 on the NGLD, PlantDoc, and PlantVillage datasets, respectively, and AUC values of 0.9995, 0.8690, and 0.9989, respectively, outperforming the comparison methods. Ablation experiments and sensitivity analysis further verify the effectiveness and stability of each module. Model complexity analysis shows that the proposed method maintains acceptable computational overhead while achieving superior recognition performance.

## 1 Introduction

Plant diseases are one of the important factors affecting crop yield, crop quality, and the stability of agricultural productionStrange and Scott (2005). Timely and accurate identification of disease types in plant leaves is of great significance for disease prevention and control, precise pesticide application, field management decision-making, and the construction of smart agriculture systems. With the development of agricultural image acquisition devices, mobile terminals, and intelligent sensing technologies, automatic plant disease recognition based on leaf images has gradually become a key task in intelligent agricultural diagnosis Savary et al. (2019); Chettri et al. (2026). Compared with traditional recognition methods that rely on manual experience, deep learning methods can automatically extract visual features such as color variations, lesion textures, edge morphology, and spatial structures from images, providing effective technical support for large-scale, low-cost, and real-time plant disease recognition.

However, existing plant disease classification methods still face several specific challenges in complex scenarios Barbedo (2016); Chettri et al. (2024). First, field-collected images usually contain interference factors such as cluttered backgrounds, uneven illumination, leaf occlusion, changes in shooting angles, and variations in lesion scales, causing models to easily mistake non-disease regions, vein textures, or background noise as discriminative cues. Second, different disease categories may present similar color abnormalities, spot morphology, and edge variations, while the same disease may show significantly different appearances at different growth stages. This makes inter-class boundaries more ambiguous and intra-class variations more complex Barbedo (2016). Third, although convolutional models can capture local textures, their ability to model cross-region disease distribution relationships is limited; although Transformer models have advantages in global modeling, they may still be insufficient in extracting fine-grained lesion textures and local boundary details Mohanty et al. (2016). Finally, existing classification models usually directly output a single class label, which can easily produce overconfident incorrect predictions on visually similar diseases, low-quality images, or distribution-shifted samples, making it difficult to meet the requirements for reliability and interpretability in practical agricultural diagnosis scenarios Ferentinos (2018).

To address the above problems, this paper constructs a reliable visual recognition framework for plant disease image classification. The framework adopts Swin-Tiny as the basic visual backbone network to extract hierarchical image semantic features. On this basis, a multifrequency selective state-space feature enhancement module is further designed to enhance disease-related evidence from three aspects: low-frequency leaf structure, high-frequency lesion texture, and cross-region state-space context. The low-frequency branch is used to preserve the overall leaf morphology, color distribution, and large-scale disease variations; the highfrequency branch is used to highlight lesion edges, local spots, and texture abnormalities; and the state-space branch is used to capture long-range dependencies among different disease regions. Meanwhile, this paper introduces a conformal-aware reliable prediction mechanism, which incorporates the idea of reliable prediction into the classification output process. In this way, while giving conventional class predictions, the model can also form candidate prediction sets with uncertainty expression capability, thereby improving the trustworthiness of plant disease recognition in complex scenarios.

The main contributions of this paper are summarized as follows:

- A multi-frequency selective state-space feature enhancement module is proposed for plant disease classification. Through the joint design of low-frequency structure modeling, highfrequency texture enhancement, and state-space contextual modeling, the module improves the ability of the model to represent fine-grained visual evidence of plant diseases.
- A conformal-aware reliable prediction mechanism is constructed. By introducing reliable coverage constraints into the plant disease recognition process, the model can further express prediction uncertainty beyond category discrimination, thereby reducing the risk of overconfident predictions on complex samples.
- A unified recognition framework is formed that balances feature discriminability, prediction reliability, and computational overhead, providing a practical and promising solution for plant disease image classification under complex backgrounds, multi-class settings, and fine-grained visual differences.

## 2 Related work

### 2.1 Deep visual models for plant disease classification

In recent years, deep visual models have become the mainstream technical route in plant disease classification research, with the core objective of automatically learning discriminative features from leaf images, including lesion texture, color abnormalities, edge morphology, and local structural variations. Atila et al. introduced EfficientNet into plant leaf disease classification at an relatively early stage, demonstrating that convolutional networks based on compound scaling can achieve a favorable balance between parameter efficiency and classification performance Atila et al. (2021). On this basis, Borhani et al. further explored the application of Vision Transformer to automated plant disease classification, showing that visual models based on self-attention mechanisms can capture long-range regional dependencies and providing an important reference for the transition of plant disease recognition from local convolutional modeling to global semantic modeling Borhani et al. (2022). Subsequently, You et al. combined SimAM-EfficientNet with adversarial attack analysis, emphasizing that model robustness and security should still be considered in addition to high-accuracy classification You et al. (2023). Nazir et al. proposed EfficientPNet for potato leaf disease classification, further demonstrating that lightweight design and structural optimization tailored to specific crop disease scenarios can improve the practical deployment value of the model Nazir et al. (2023). Zeng et al. integrated disease classification with image description generation through DIC-Transformer, indicating that plant disease recognition has begun to move beyond classification accuracy toward result interpretation and semantic expression Zeng et al. (2024).

Although the above studies have promoted the continuous development of plant disease classification models from traditional CNNs to Transformers, hybrid architectures, and interpretable frameworks, existing methods still have several limitations. Salman et al. introduced Vision Transformer and mixture of experts for plant disease recognition in field environments, indicating that complex backgrounds, illumination variations, and intra-class differences in real-world scenarios can still significantly affect model generalization Salman et al. (2025). Yu et al. proposed ST-CFI, which enhances local and global representations through feature interaction between Swin Transformer and convolutional features, suggesting that relying solely on Transformer-based global modeling may be insufficient to fully capture fine-grained lesion textures Yu et al. (2025). Liu et al. designed a plant disease detection algorithm based on Efficient Swin Transformer, further reflecting the potential of hierarchical Transformer structures in visual disease recognition; however, its performance improvement still mainly depends on a stronger backbone architecture Liu and Zhang (2025). Krishna et al. validated the applicability of deep learning methods for plant leaf disease detection from a multi-dataset perspective, showing that data distribution differences and class imbalance remain challenging in cross-dataset scenarios Krishna et al. (2025). Jawed et al. improved the robustness of disease classification through a hybrid framework combining convolutional models and Transformer models, reflecting the complementary value of local texture modeling and global dependency modeling Jawed et al. (2026). Batool et al. further improved the plant leaf disease classification capability of Vision Transformer using a multi-patch selection mechanism; however, such methods still mainly focus on discriminative feature extraction, while paying insufficient attention to trustworthy diagnostic issues such as model prediction uncertainty, reliable coverage, and candidate class sets Batool et al. (2026). Therefore, although existing deep visual models have made significant progress in classification accuracy, there remains further research space in fine-grained disease evidence enhancement under complex backgrounds, cross-region lesion dependency modeling, and reliable prediction output.

### 2.2 Reliable disease recognition with feature enhancement and conformal prediction

Reliable plant disease recognition depends not only on classification accuracy, but also on the ability of models to form more stable disease evidence representations under complex backgrounds, fine-grained lesions, and visually similar categories. Around feature enhancement, recent studies have improved visual representations for plant disease recognition from the perspectives of frequency-domain decomposition, spatial-domain fusion, and state-space modeling. Lin et al. introduced plant disease recognition into a frequency-domain few-shot learning framework, showing that frequency-domain information can compensate for the limitations of traditional spatial texture features in small-sample disease recognition Lin et al. (2022). Li et al. further proposed a dual-branch network that integrates frequency-domain and spatial-domain information, indicating that low-frequency structural information and highfrequency texture information have complementary value in crop disease recognition Li et al. (2024). DWTFormer, proposed by Xiang et al., uses discrete wavelet transform and spatial feature fusion to enhance tomato leaf disease recognition, providing a direct reference for the joint modeling of lesion edges, texture details, and overall leaf structure Xiang et al. (2025). Zhao et al. adopted a wavelet-based frequency-domain method for multi-crop disease detection, further demonstrating that frequency-domain features have certain generalization potential in cross-crop disease recognition Zhao et al. (2026). Meanwhile, Vision Mamba proposed by Zhu et al. uses a bidirectional state-space model for efficient visual representation learning, providing a new pathway for long-range dependency modeling that differs from self-attention mechanisms Zhu et al. (2024). VMamba, proposed by Liu et al., enhances two-dimensional image modeling through a visual state-space structure, showing that the selective scanning mechanism can capture spatial contextual relationships while maintaining high efficiency Liu et al. (2024). Zhang et al. applied VMamba to plant leaf disease recognition, demonstrating the application value of state-space models in agricultural image classification tasks Zhang et al. (2025). Mamun et al. further explored progressive self-supervised Vision Mamba for plant disease recognition, indicating that the combination of state-space models and self-supervised representation learning can help alleviate the problem of insufficient annotated data Mamun et al. (2025). However, existing frequency-domain enhancement methods mainly focus on the fusion of frequency components, while state-space methods mainly emphasize global dependency modeling. These two types of methods are often used independently and have not yet fully addressed the unified enhancement of low-frequency leaf structures, high-frequency lesion textures, and cross-region disease distribution relationships in plant disease images. Therefore, combining multi-frequency feature decomposition with selective state-space modeling can further highlight disease-related evidence after the backbone network and reduce the interference of background textures and non-disease regions in classification decisions.

In addition to feature representation, reliable disease recognition also needs to address the overconfidence problem commonly observed in deep classification models. Traditional softmax outputs usually provide only a single class probability, but this probability is not equivalent to statistical confidence, especially under field environments, visually similar disease categories, and data distribution shifts, where models may produce high-confidence incorrect predictions. Angelopoulos et al. introduced conformal prediction into uncertainty set construction for image classifiers, enabling models to output prediction sets containing one or more candidate classes instead of only a single label Angelopoulos et al. (2020). Bates et al. systematically introduced the basic ideas of conformal prediction and distribution-free uncertainty quantification, emphasizing that it can provide coverage guarantees under relatively weak distributional assumptions Angelopoulos and Bates (2021). RR-CP, proposed by Zhang et al., introduced the idea of reliable regions into conformal prediction for medical image classification, showing that prediction sets can be used to improve the trustworthiness of high-risk image recognition tasks Zhang et al. (2023). Fayyad et al. validated the effectiveness of conformal prediction in skin lesion classification, indicating that this method can provide more reliable decision support for medical image models Fayyad et al. (2024). Zhang et al. evaluated the practicality of conformal prediction sets from the perspective of human-AI collaborative image annotation, suggesting that prediction sets are not only statistical tools, but can also influence users’ understanding and adoption of model suggestions Zhang et al. (2024). Penso et al. further investigated noise-robust conformal prediction, pointing out that reliable prediction sets still need to be combined with more robust calibration mechanisms when label noise or data perturbations are present Penso and Goldberger (2024). However, most existing conformal prediction studies treat it as a post-training calibration module, with limited integration into the disease feature learning process. For plant disease classification tasks, generating prediction sets only at the inference stage cannot directly improve the separability of disease evidence in the feature space. Therefore, it is necessary to further incorporate the reliable coverage concept of conformal prediction into the training objective, so that the model can focus on true-class inclusion, prediction set compactness, and uncertainty expression while optimizing classification discriminability. In this way, it can form a complementary relationship with the multi-frequency selective state-space feature enhancement module: the former improves the quality of disease evidence representation, while the latter constrains the reliability of model outputs and the interpretability of decision boundaries.

## 3 Method

### 3.1 Overall model architecture

**Figure 1:**
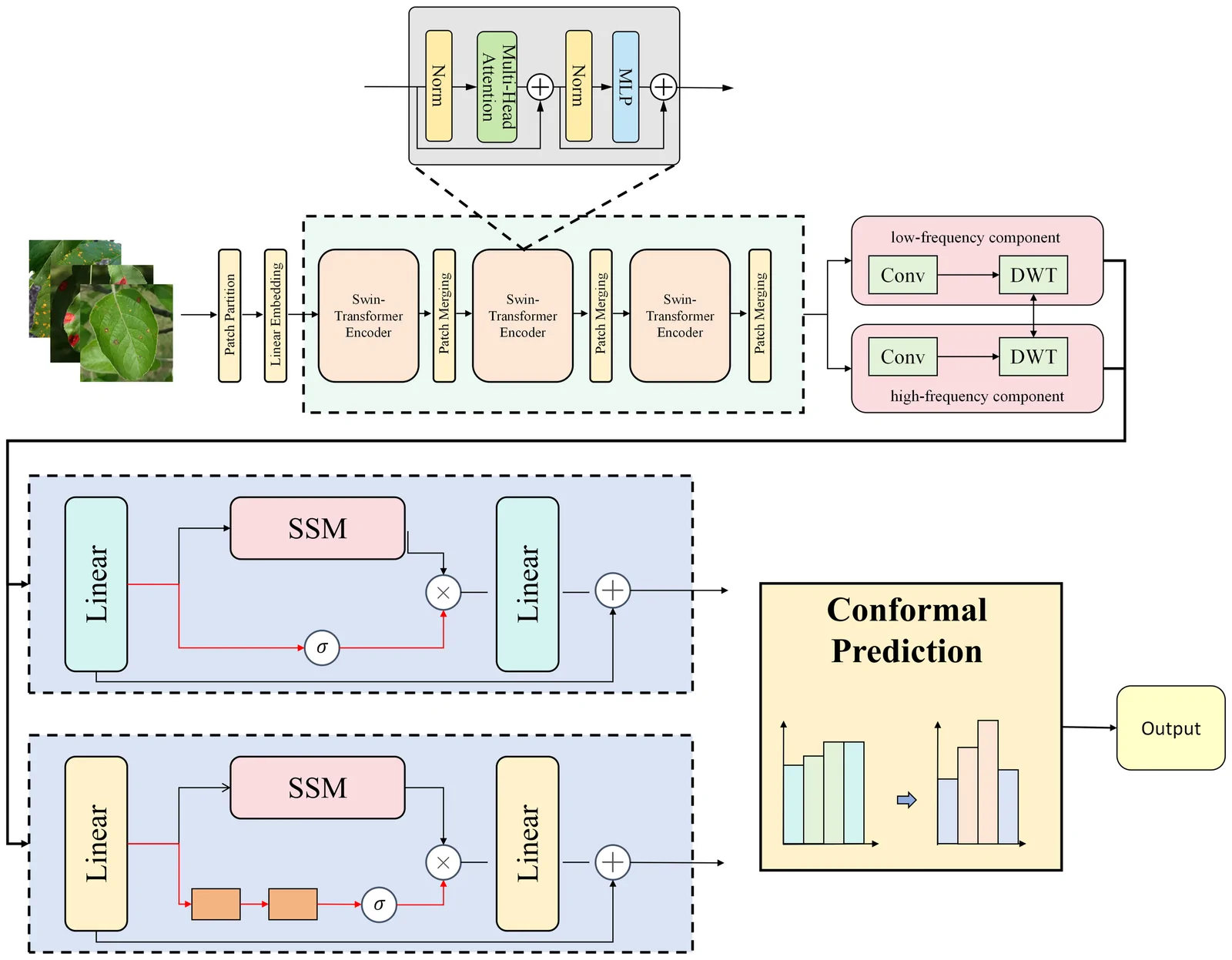
Schematic illustration of the overall architecture of the proposed model, in which the Swin-Tiny backbone network is used for hierarchical visual feature extraction, the MF-SSFE module is used for multi-frequency disease feature enhancement, and the Conformal Prediction module is used to generate reliable disease prediction sets.

This paper constructs a reliable visual recognition framework for plant disease classification, which consists of a Swin-Tiny backbone network, a multi-frequency selective state-space feature enhancement module, and a conformal prediction module. Given an input leaf image $x\;\in \;{\mathbb{R}}^{H\times W\times 3}$, image preprocessing and patch embedding are first performed to map it into visual token representations, which are then fed into multi-layer Swin Transformer Encoders to extract hierarchical visual features. Swin-Tiny models fine-grained texture relationships within local regions through window-based self-attention and the shifted-window mechanism, and forms higher-level semantic representations through progressive patch merging. Different from directly using a classification head for prediction, this paper further introduces a multi-frequency selective state-space feature enhancement module after the backbone output to simultaneously strengthen low-frequency leaf structures, high-frequency lesion textures, and cross-region disease distribution relationships. The overall model architecture is shown in **Figure 1**.

The overall feature flow can be formulated as:

(1) $$F_{s}={\text{Φ}}_{\text{swin}}(x),\;F_{e}={\text{Φ}}_{\text{MF}-\text{SSFE}}(F_{s}),\;z=\text{GAP }(F_{e})$$

As shown in Equation 1, where Φ_swin_(·) denotes the Swin-Tiny backbone network, **F***_s_*denotes the semantic features extracted by the backbone, Φ_MF-SSFE_(·) denotes the multi-frequency selective state-space feature enhancement module, **F***_e_*denotes the enhanced disease representation, GAP(·) denotes the global average pooling operation, and **z** denotes the image-level feature finally used for classification and reliability estimation. This design enables the model to rely not only on the hierarchical semantic modeling capability of Transformer, but also to explicitly enhance key disease evidence before classification, including lesion edges, spot textures, mildew details, and overall structural changes of leaves, thereby improving the discriminative ability of the model under complex backgrounds and visually similar disease categories.

In the feature enhancement stage, the MF-SSFE module first performs multi-frequency decomposition on the backbone output features and divides them into low-frequency structural components and high-frequency detail components. The low-frequency component is used to preserve the overall leaf morphology, color distribution, and large-scale disease variations, while the high-frequency component is used to highlight lesion boundaries, local spots, and texture abnormalities. Subsequently, the selective state-space branch serializes and scans the features along the horizontal and vertical directions to capture long-range dependencies and spatial diffusion relationships among disease regions. The three branches are fused through an adaptive gating mechanism, enabling the model to dynamically adjust the contributions of low-frequency structure, high-frequency texture, and state-space context according to different samples. This process can be expressed as:

(2) $$F_{e}=F_{s}+\lambda (\omega_{l}F_{l}+\omega_{h}F_{h}+\omega_{m}F_{m})$$

As shown in Equation 2, where **F***_l_*denotes the low-frequency structural features, **F***_h_*denotes the high-frequency disease texture features, **F***_m_*denotes the long-range contextual features extracted by the selective statespace branch, *ω_l_*, *ω_h_*, and *ω_m_*denote the adaptive fusion weights, and *λ* denotes the residual enhancement coefficient. The enhanced image-level feature **z** is then fed into the classification head to obtain the class probability distribution, and is further input into the conformal prediction module to generate a reliable prediction set. Unlike traditional classification methods that output only a single class label, the conformal prediction module constructs a candidate class set based on calibration scores and a quantile threshold, allowing the model to output multiple possible disease categories for uncertain samples and thereby reducing high-confidence incorrect predictions. The final prediction set is defined as:

(3) $$C_{\alpha }(x)=\{y\in Y\;|\text{ s}1-p(y|x)\leq q_{1-\alpha }\}$$

As shown in Equation 3, where Y denotes the entire disease class space, p(y|x) denotes the predicted probability of class *y* given by the model, *α* denotes the significance level, and *q*_1−_*_α_*denotes the quantile threshold of the nonconformity scores obtained from the calibration samples. By combining the disease evidence enhancement capability of MF-SSFE with the reliable set output of conformal prediction, the proposed framework can improve the quality of classification representations while providing more trustworthy prediction results.

### 3.2 Multi-frequency selective state-space feature enhancement module

To enhance the representation of fine-grained lesion textures, overall leaf structures, and cross-region disease distribution relationships in plant disease images, this paper designs a Multi-Frequency Selective State-Space Feature Enhancement Module, abbreviated as MF-SSFE, after the Swin-Tiny backbone network. Given the intermediate feature $F\in {\mathbb{R}}^{C\times H\times W}$ output by the backbone network, this module first divides the feature into one low-frequency sub-band and three high-frequency sub-bands through two-dimensional Haar discrete wavelet transform. The decomposition is implemented by non-overlapping 2 × 2 spatial sampling with a stride of 2 and does not introduce additional learnable parameters. Therefore, each decomposed sub-band has a spatial size of $\frac{H}{2}\times \frac{W}{2}$ while retaining the original channel dimension *C*. The low-frequency sub-band mainly preserves the overall leaf color, contour structure, and large-scale disease variations, while the high-frequency sub-bands highlight detailed information such as lesion edges, local spots, mildew textures, and necrotic region boundaries. Specifically, let **F**_00_, **F**_01_, **F**_10_, and **F**_11_ denote the four sub-features obtained by sampling **F** according to odd-even spatial positions. The wavelet decomposition process can be formulated as:

(4) $$\left\{ \begin{array}{ll} F_{LL} & =\frac{1}{2}(F_{00}+F_{01}+F_{10}+F_{11})\\ F_{LH} & =\frac{1}{2}(F_{00}-F_{01}+F_{10}-F_{11})\\ F_{HL} & =\frac{1}{2}(F_{00}+F_{01}-F_{10}-F_{11})\\ F_{HH} & =\frac{1}{2}(F_{00}-F_{01}-F_{10}+F_{11}) \end{array}\right.$$

As shown in Equation 4, where **F***_LL_*denotes the low-frequency structural component, and **F***_LH_*, **F***_HL_*, and **F***_HH_*denote the high-frequency detail components in different directions. Subsequently, the low-frequency branch and the high-frequency branch reconstruct features through convolutional mappings, respectively. The high-frequency branch first concatenates the three high-frequency sub-bands along the channel dimension and then compresses them back to the original channel dimension through a nonlinear transformation. Specifically, the low-frequency branch applies a 3 × 3 convolution with *C* input and output channels, followed by normalization and GELU activation. In the high-frequency branch, the concatenated 3*C*-channel representation is first compressed to *C* channels by a 1 × 1 convolution and is then refined by a 3 × 3 convolution, normalization, and GELU activation. This process is defined as:

(5) $$F_{l}=U(\psi_{l}(F_{LL})),\;F_{h}=U(\psi_{h}(\text{Concat }[F_{LH},F_{HL},F_{HH}]))$$

As shown in Equation 5, where *ψ_l_*(·) and *ψ_h_*(·) denote the convolution-normalization-nonlinear mappings in the lowfrequency branch and the high-frequency branch, respectively, and U(·) denotes the bilinear upsampling operation, which is used to restore the low-frequency and high-frequency features to the same spatial resolution as the input feature. Bilinear interpolation is performed with the target size explicitly set to *H* ×*W*, and the channel dimension remains unchanged during spatial reconstruction. Through this design, the model can simultaneously maintain the global stability of low-frequency structures and the local sensitivity of high-frequency lesion textures, avoiding excessive attention to non-disease regions by single spatial-domain features under complex backgrounds.

In addition to multi-frequency decomposition, MF-SSFE further introduces a selective statespace modeling branch to capture long-range dependencies of disease regions on the leaf surface. Different from local convolution, which mainly focuses on neighborhood textures, the state-space branch performs selective recurrent scanning of two-dimensional features along the horizontal and vertical directions, enabling lesion responses at different positions to establish contextual connections through state propagation. For the input feature **F**, the state-space input is first obtained by linear projection as **X** = **W***_x_*∗ **F**, where ∗ denotes convolutional mapping. In implementation, **W***_x_*is a 1 × 1 convolution that maps the input from *C* channels to a state dimension of *C*, without additional channel expansion. The projected feature is reshaped into horizontal sequences of length *W* and vertical sequences of length *H*. Four directional scans are then performed, including left-to-right, right-to-left, top-to-bottom, and bottom-to-top scanning. Each direction uses an independently learnable channel-wise state retention vector, enabling different propagation behaviors for different spatial orientations. Taking horizontal scanning as an example, the hidden state at the *t*-th spatial position is updated as:

(6) $$s_{t}^{h}=\alpha_{h}⨀s_{t-1}^{h}+(1-\alpha_{h})⨀x_{t}^{h},\;\alpha_{h}=\sigma (a_{h})$$

As shown in Equation 6, where **s***^ht^*denotes the state representation at the *t*-th position in the horizontal direction, **x***^ht^*denotes the input feature at the corresponding position, *α_h_*is a learnable state retention coefficient, ⊙ denotes element-wise multiplication, and *σ*(·) denotes the Sigmoid function. The initial state of each sequence is set to zero, and the retention coefficient is constrained to (0,1) by the Sigmoid function to maintain stable recurrent propagation. Similarly, the same form of state recurrence is also performed in the vertical direction. To avoid directional bias caused by unidirectional scanning, this paper uses both forward and backward scanning, and aggregates the state responses in the horizontal and vertical directions:

(7) $$F_{m}=\phi_{m}(\frac{{\vec{S}}_{h}+{\overset{\leftarrow }{S}}_{h}+{\vec{S}}_{v}+{\overset{\leftarrow }{S}}_{v}}{4})$$

As shown in Equation 7, where ${\vec{S}}_{h}$ and ${\overset{\leftarrow }{S}}_{h}$ denote the forward and backward state responses in the horizontal direction, respectively, ${\vec{S}}_{v}$ and ${\overset{\leftarrow }{S}}_{v}$denote the forward and backward state responses in the vertical direction, respectively, and *ϕ_m_*(·) denotes the state-space output mapping. The four directional responses are restored to the original two-dimensional arrangement before aggregation, and *ϕ_m_*(·) is implemented by a 1×1 convolution followed by normalization, producing the contextual feature $F_{m}\in {\mathbb{R}}^{C\times H\times W}$. The detailed computation process is shown in Algorithm 1.

**Table:** 

Algorithm 1 Forward propagation process of the MF-SSFE module.
**Require:** Backbone output feature $F\in {\mathbb{R}}^{C\times H\times W}$ **Ensure:** Enhanced disease feature **F***_e_* 1: Perform Haar-DWT decomposition on **F** to obtain **F***_LL_*, **F***_LH_*, **F***_HL_*, and **F***_HH_* 2: Feed **F***_LL_*into the low-frequency branch to obtain the low-frequency structural feature **F***_l_* 3: Concatenate **F***_LH_*, **F***_HL_*, and **F***_HH_*, and feed them into the high-frequency branch to obtain the high-frequency texture feature **F***_h_* 4: Perform horizontal forward-backward and vertical forward-backward selective state-space scanning on the input feature **F** to obtain the long-range contextual feature **F***_m_*5: Concatenate **F***_l_*, **F***_h_*, and **F***_m_*to compute the adaptive gating weights *ω_l_*, *ω_h_*, and *ω_m_* 6: Fuse multi-branch features according to the gating weights to obtain **F***_a_* 7: Obtain the final enhanced feature through residual connection: **F***_e_*= **F** + *λ***F***_a_* 8: **return F***_e_*

In the feature fusion stage, MF-SSFE does not directly add the low-frequency, high-frequency, and state-space features in a simple manner, but dynamically estimates the importance of different branches through an adaptive gating mechanism. For different disease categories, the visual evidence relied on by the model is not the same. For example, mildew diseases usually rely more on high-frequency textures and local spots, leaf spot diseases rely more on boundary morphology and regional distribution, while healthy leaves or large-scale lesions rely more on low-frequency structures and overall color variations. Therefore, this paper generates spatially adaptive fusion weights based on the joint representation of the three types of features:

(8) $$\left[\omega_{l},\omega_{h},\omega_{m}]=\text{Softmax }(W_{g}*\delta (W_{f}*\text{Concat }[F_{l},F_{h},F_{m}])\right)$$

As shown in Equation 8, where **W***_f_* and **W***_g_* are learnable convolutional parameters, *δ*(·) denotes the nonlinear activation function, and *ω_l_*, *ω_h_*, and *ω_m_*denote the fusion weights of low-frequency structure, highfrequency texture, and state-space context, respectively, satisfying *ω_l_*+ *ω_h_*+ *ω_m_*= 1. More specifically, the concatenated feature with 3*C* channels is first projected to *C/*4 channels by the 1 × 1 convolution **W***_f_*, followed by GELU activation. The gating projection **W***_g_*then maps the reduced feature to three spatial weight maps of size *H* × *W*. Softmax normalization is applied along the branch dimension at every spatial position so that the three branch weights sum to one locally rather than only at the image level. Finally, the enhanced feature is obtained through gated weighting and residual injection:

(9) $$F_{e}=F+\lambda \cdot \rho (\omega_{l}⨀F_{l}+\omega_{h}⨀F_{h}+\omega_{m}⨀F_{m})$$

As shown in Equation 9, where *ρ*(·) denotes the feature refinement mapping, and *λ* is a learnable residual scaling coefficient. The refinement mapping *ρ*(·) consists of a 3 × 3 convolution, normalization, and GELU activation, with both the input and output channel dimensions set to *C*. The residual scaling coefficient *λ* is initialized to a small value and jointly optimized with the remaining network parameters, allowing the enhanced representation to be progressively injected into the backbone feature during training. This residual design can prevent the enhancement module from disrupting the semantic representations already learned by the backbone network in the early training stage, while allowing the model to gradually learn a more effective disease evidence enhancement strategy. By integrating multi-frequency feature decomposition, selective statespace modeling, and adaptive gated fusion into the same module, MF-SSFE can enhance the responses of disease-related regions while maintaining a compact model structure, and improve the discriminability and stability of the features received by the subsequent classification head and conformal prediction module.

### 3.3 Conformal-aware reliable prediction

**Figure 2:**
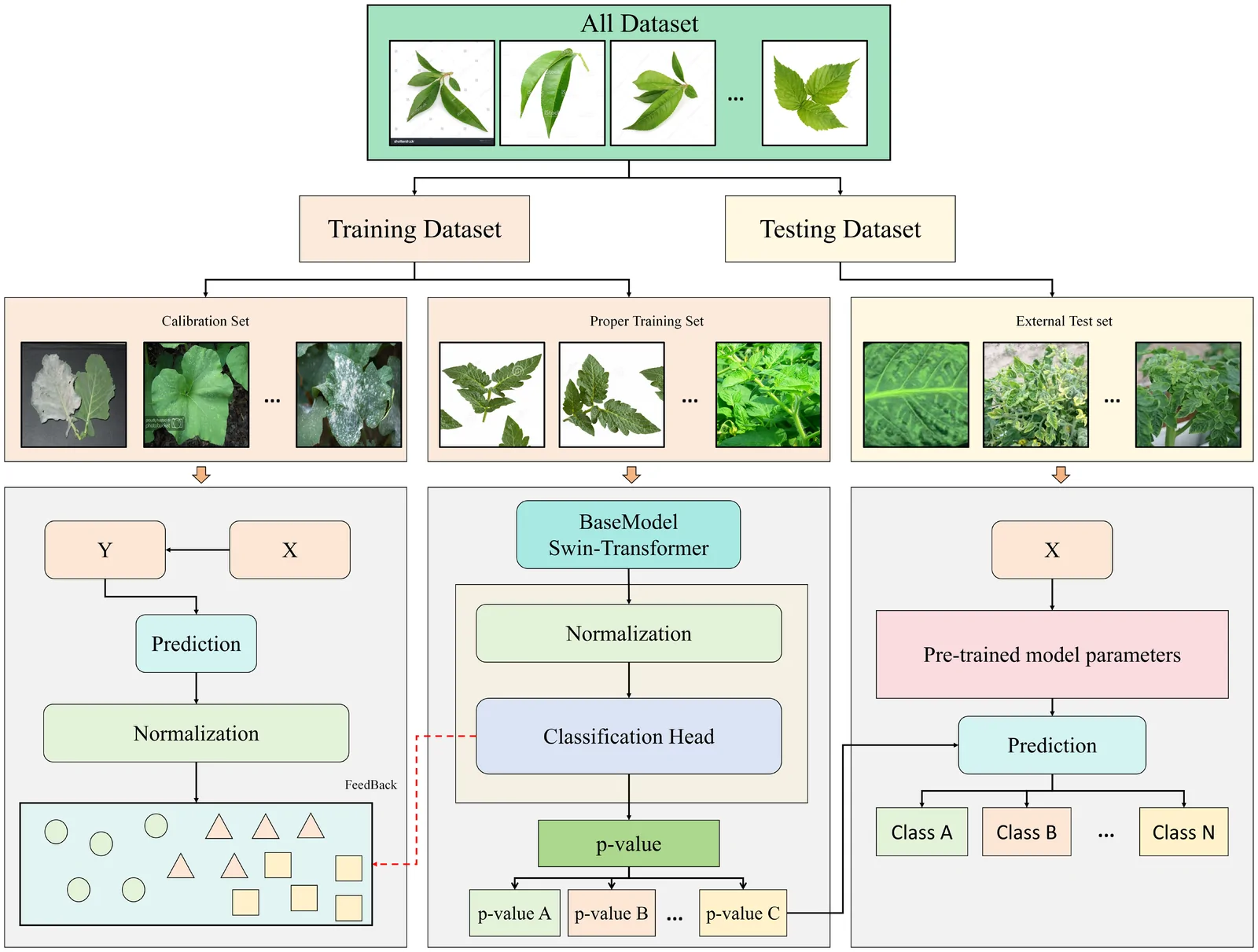
Schematic illustration of the overall workflow of the conformal-aware reliable prediction module, in which the training data are divided into a calibration set and a proper training set. The calibration set is used to estimate the conformal threshold and class-level *p*values, while the external test set is finally used to generate reliable prediction results containing multiple candidate classes.

After enhancement by the MF-SSFE module, the model obtains image-level features that simultaneously contain low-frequency leaf structures, high-frequency lesion textures, and longrange disease contexts. To avoid overconfident predictions caused by traditional classification models that output only a single class label, this paper further designs a conformal-aware reliable prediction module. This module maps the enhanced features into a class probability distribution and introduces the idea of conformal prediction during both training and inference, enabling the model not only to learn class-discriminative boundaries but also to form candidate disease sets with coverage constraints. The architecture of this module is shown in **Figure 2**.

Given the enhanced feature **F***_e_*output by MF-SSFE, global pooling and normalization are first performed to obtain the image-level representation **z**, and then the classification head generates logits and the class probability distribution:

(10) $$z=\text{LN }(\text{GAP }(F_{e})),\;l=W_{c}z+b_{c},\;p_{k}(x)=\frac{\exp\;(l_{k}/\tau )}{\sum_{j=1}^{K}\exp\;(l_{j}/\tau )}$$

As shown in Equation 10, where LN(·) denotes layer normalization, GAP(·) denotes global average pooling, *ℓ* denotes the classification logits, *K* denotes the number of classes, *τ* denotes the temperature coefficient, and *p_k_*(*x*) denotes the predicted probability that the input sample *x* belongs to the *k*-th disease class. This probability distribution is used for ordinary classification prediction on the one hand, and is also fed into the conformal prediction module to construct nonconformity scores on the other hand. Different from directly using the maximum softmax probability as confidence, this paper jointly uses the true-class probability and the competing-class probabilities to characterize the nonconformity degree of a sample, thereby more fully reflecting the discriminative uncertainty of the model among visually similar disease categories:

(11) $$s(x,y)=(1-p_{y}(x))+\beta \cdot \log\;(1+\overset{K}{\underset{\begin{array}{c} j=1\\ j\neq y \end{array}}{\sum }}\exp(\frac{p_{j}(x)-p_{y}(x)}{\gamma }))$$

As shown in Equation 11, where s(x,y) denotes the nonconformity score of sample *x* under class *y*, *p_y_*(*x*) denotes the predicted probability of class *y*, the second term is used to characterize the competition intensity of non-true classes against the true class, *β* denotes the weight of the competition term, and *γ* denotes the smoothing coefficient. When the true-class probability is low or multiple competing classes have probabilities close to the true-class probability, the nonconformity score increases, indicating that the model has stronger uncertainty in judging the disease category of the sample.

As shown in the figure, the conformal prediction module contains three key parts: the proper training set, the calibration set, and the external test set. The proper training set is used to learn the parameters of the backbone network, the MF-SSFE module, and the classification head; the calibration set is used to estimate the conformal threshold; and the external test set is used to generate the final reliable prediction set. Let the calibration set be $D_{cal}={\left\{(x_{i},y_{i})\right\}}_{i=1}^{n}$. The calibration samples are input into the trained model to obtain the set of calibration nonconformity scores $S_{cal}$:

(12) $$S_{cal}={\left\{s_{i}=s(x_{i},y_{i})|(x_{i},y_{i})\in D_{cal}\right\}}_{i=1}^{n}$$

As shown in Equation 12, Then, the scores in $S_{cal}$are sorted in ascending order, and the quantile threshold with finitesample correction is calculated according to the significance level *α*:

(13) $$q_{\alpha }=s_{\left(k\right)},\;k=[(n+1)(1-\alpha )],\;s_{\left(1\right)}\leq s_{\left(2\right)}\leq \cdots \leq s_{\left(n\right)}$$

As shown in Equation 13, where *q_α_* denotes the conformal calibration threshold, and a smaller *α* indicates that the model needs to provide a prediction set with a higher coverage level. For a test sample *x* and any candidate class *y*, the conformal module further calculates the class-level *p*-value to measure the consistency between this class and the calibration distribution:

(14) $$\pi_{y}(x)=\frac{1+\sum_{i=1}^{n}I[s_{i}\geq s(x,y)]}{n+1}$$

As shown in Equation 14, where *π_y_*(*x*) denotes the conformal *p*-value of class *y*, and I[·] denotes the indicator function. When the nonconformity score corresponding to a class is no larger than the scores of most calibration samples, this class obtains a higher *p*-value, indicating that it is more consistent with the calibration data distribution. Finally, the reliable prediction set of the test sample consists of all classes that pass the significance test:

(15) $$C_{\alpha }(x)=\{y\in Y|\pi_{y}(x)>\alpha \}=\{y\in Y|s(x,y)\leq q_{\alpha }\}$$

As shown in Equation 15, where $C_{\alpha }(x)$ denotes the conformal prediction set under the significance level *α*, and Y denotes the entire class space of plant diseases. When the model has high certainty for a sample, $C_{\alpha }(x)$ usually contains only one class. When lesion appearances are similar, background interference is strong, or the sample is located near the class boundary, the prediction set may contain multiple candidate classes, thereby avoiding the forced output of a single but unreliable disease label.

To make conformal prediction not merely a post-processing operation in the inference stage, this paper further incorporates the idea of conformal reliability into the training stage, so that the model can focus on true-class coverage and prediction set compactness while optimizing classification discriminability. In each training batch, the batch-level nonconformity threshold ${\hat{q}}_{B}$is estimated according to the current probability distribution, and a differentiable soft set membership function is used to approximate whether a class is included in the prediction set:

(16) $$m_{k}(x)=\sigma (\frac{{\hat{q}}_{B}-s(x,k)}{\delta })$$

(17) $${ℛ}_{cp}={\left[\left(1-\alpha \right)-\frac{1}{B}\sum_{i=1}^{B}m_{y_{i}}\left(x_{i}\right)\right]}_{+}+\eta \cdot \frac{1}{B}\sum_{i=1}^{B}\left|C_{i}\right|$$

As shown in Equation 16, Equation 17, where *m_k_*(*x*) denotes the degree to which class *k* is softly included in the prediction set, *δ* denotes the smoothing temperature, *B* denotes the batch size, the first term encourages the true class to be included in the prediction set and approach the target coverage level, the second term constrains the average prediction set size, and *η* is used to balance coverage and compactness. Through this conformal-aware constraint, the model can reduce high-confidence incorrect predictions during the training stage and learn a more stable probability distribution. Finally, the model output includes not only the ordinary top-1 disease class, but also the conformal prediction set and the uncertainty measure:

(18) $$O(x)=(\hat{y},C_{\alpha }(x),u(x)),\;\hat{y}=\arg\;\underset{y\in Y}{\max}\;p_{y}(x),\;u(x)=\frac{\left|C_{\alpha }(x)\right|}{K}$$

As shown in Equation 18, where $\hat{y}$ denotes the conventional maximum-probability predicted class, $C_{\alpha }(x)$ denotes the reliable candidate disease set, and *u*(*x*) denotes the uncertainty indicator obtained by normalizing the set size. This module forms a complementary relationship with the MF-SSFE module introduced in the previous section: MF-SSFE is responsible for enhancing disease-related visual evidence, while the conformal-aware reliable prediction module constrains the trustworthiness and set-based expression capability of model outputs. Therefore, the proposed method can simultaneously consider classification accuracy, prediction reliability, and practical diagnostic safety in complex plant disease images.

## 4 Experimental results and analysis

### 4.1 Experimental setup

All experiments were conducted under a unified software and hardware environment and consistent training configurations. The experiments were implemented using the PyTorch deep learning framework. Swin-Tiny was adopted as the backbone model, and all input images were uniformly resized to 224 × 224. The Swin-Tiny backbone was initialized using weights pre-trained on ImageNet-1K and subsequently fine-tuned on each plant disease dataset. For NGLD, PlantDoc, and PlantVillage, the samples were stratified by category and divided into a proper training set, calibration set, validation set, and test set using proportions of 70%, 10%, 10%, and 10%, respectively. The same fixed data partitions were used for all compared methods to ensure a fair comparison, and the calibration samples were excluded from model training and used only to estimate the conformal threshold. All experiments were independently repeated using three random seeds, and the results are reported as the mean and standard deviation across the three runs. AdamW was used as the optimizer, with the initial learning rate set to 1 × 10^−4^ and the weight decay coefficient set to 1 × 10^−4^. The learning rate was scheduled using cosine annealing decay. All models were trained for 50 epochs with a batch size of 32, and automatic mixed precision training was adopted to reduce GPU memory consumption and improve training efficiency. The specific software and hardware environment and main training hyperparameters are shown in **Table 1**.

**Table 1:** Experimental environment and main training hyperparameter settings.

Item	Setting
Operating system	Ubuntu Linux
Programming language	Python 3.10
Deep learning framework	PyTorch
Vision model libraries	timm, torchvision
GPU	4090D
Backbone network	Swin-Tiny
Backbone initialization	ImageNet-1K pre-trained weights
Input image size	224 × 224
Data split	Proper training/calibration/validation/test: 70%/10%/10%/10%
Training epochs	50
Batch size	32
Optimizer	AdamW
Initial learning rate	1 × 10^−4^
Weight decay	1 × 10^−4^
Learning rate scheduler	CosineAnnealingLR
Loss function	Cross-Entropy Loss/Conformal-Aware Loss
Conformal significance level *α*	0.10
CP regularization weight	0.20
Set-size regularization weight	0.02

### 4.2 Evaluation metric

This paper adopts Accuracy, Precision, Recall, and AUC as model performance evaluation metrics to comprehensively evaluate model effectiveness from the perspectives of overall classification correctness, reliability of positive-class prediction, completeness of positive-class recognition, and classification ranking ability. Let *TP*, *TN*, *FP*, and *FN* denote true positives, true negatives, false positives, and false negatives, respectively.

Accuracy measures the proportion of correctly predicted samples among all samples, As shown in Equation 19, and is calculated as:

(19) $$\text{Accuracy}=\frac{TP+TN}{TP+TN+FP+FN}$$

Precision measures the proportion of true positive samples among the samples predicted as positive, As shown in Equation 20, and is calculated as:

(20) $$\text{Precision}=\frac{TP}{TP+FP}$$

Recall measures the proportion of true positive samples that are correctly identified by the model, As shown in Equation 21, and is calculated as:

(21) $$\text{Recall}=\frac{TP}{TP+FN}$$

AUC measures the overall ability of the model to distinguish samples from different classes under different classification thresholds, As shown in Equation 22, and is calculated as:

(22) $$\text{AUC}=\int_{0}^{1}\text{TPR}({\text{FPR}}^{-1}(t))dt$$

where TPR denotes the true positive rate and FPR denotes the false positive rate, As shown in Equation 23, which are defined as:

(23) $$\text{TPR}=\frac{TP}{TP+FN},\;\text{FPR}=\frac{FP}{FP+TN}$$

### 4.3 Dataset introduction

**Figure 3:**
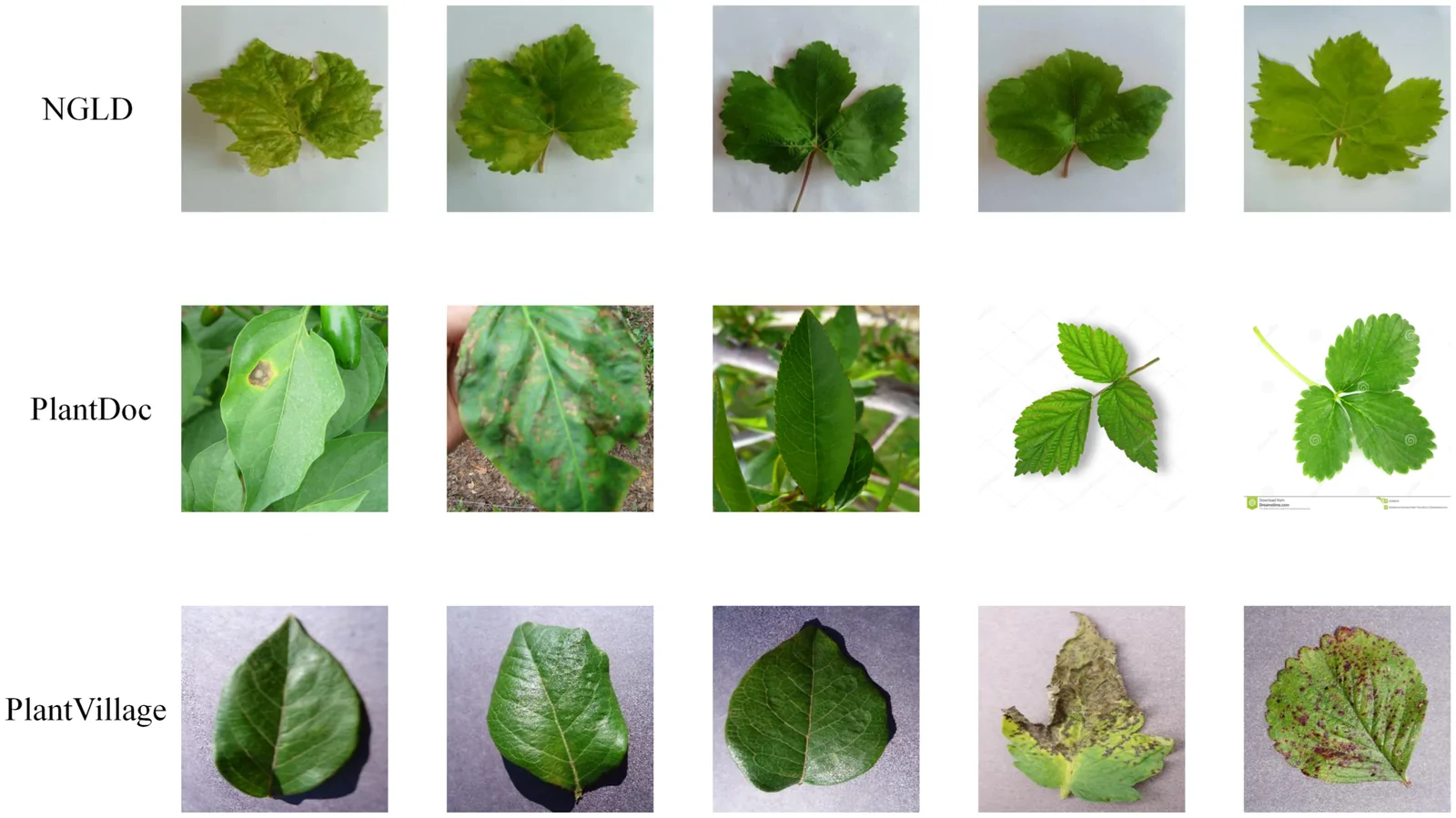
Dataset Example.

This paper selects three public plant disease image classification datasets, namely NGLD, PlantDoc, and PlantVillage, for experimental validation, so as to comprehensively evaluate the recognition ability of the model under different crop types, category scales, and image acquisition conditions. Among them, the NGLD dataset is mainly designed for grape leaf disease recognition and contains four categories, namely Bacterial-Leaf-Spot, Downy-Mildew, Healthy-Leaves, and Powdery-Mildew. It can be used to evaluate the classification performance of the model in a single-crop multi-disease scenario. The PlantDoc dataset contains leaf images of multiple crops and involves 28 categories, covering healthy leaves and typical diseased leaves of crops such as apple, tomato, corn, grape, and potato. Its image backgrounds are more complex, which can better examine the generalization ability of the model in real natural scenes. The PlantVillage dataset contains 38 categories, covering multiple crops and their common disease types. Its image backgrounds are relatively regular and its category annotations are clear, making it suitable as a dataset for evaluating plant disease classification performance under controlled environments. Experiments on the above three datasets comprehensively verify the effectiveness of the proposed method from the perspectives of single-crop disease recognition, multi-crop recognition in real-world scenes, and controlled-background classification. Dataset examples are shown in **Figure 3**.

### 4.4 Experimental results compared with other models

To verify the effectiveness of the proposed method in plant disease classification tasks, this paper selects several representative deep learning models as comparison methods and conducts unified experiments on the NGLD, PlantDoc, and PlantVillage datasets. The comparison models cover traditional convolutional neural networks, Transformer architectures, attention-enhanced models, and feature fusion methods recently used for plant disease recognition, enabling the classification ability and generalization performance of the model to be evaluated from different perspectives. All methods are compared under the same data split, input size, and evaluation metrics to ensure the fairness and comparability of the experimental results. The parameter counts and inference times are reported using approximate values because the exact computational overhead may vary slightly with dataset-specific classification heads and the corresponding number of output categories. Reporting excessively precise values could therefore be misleading, whereas approximate values provide a more appropriate representation of the computational scale of each model. The experimental results on NGLD and PlantVillage are first presented in **Tables 2**, **3**.

**Table 2:** Comparison results of different models on the NGLD dataset.

Method	Accuracy	Precision	Recall	AUC	Parameters (M)	Inference time (ms)
ResNet50He et al. (2016)	0.9124 ± 0.0067	0.9018 ± 0.0081	0.8942 ± 0.0093	0.9617 ± 0.0049	≈25	≈15.2
Vision-TransformerDosovitskiy et al. (2020)	0.9438 ± 0.0054	0.9361 ± 0.0068	0.9287 ± 0.0075	0.9824 ± 0.0036	≈112	≈6.7
RIC-NetZhao et al. (2022)	0.9576 ± 0.0048	0.9493 ± 0.0059	0.9451 ± 0.0064	0.9871 ± 0.0028	≈74	≈12.5
APDCBera et al. (2024)	0.9489 ± 0.0061	0.9417 ± 0.0063	0.9365 ± 0.0071	0.9842 ± 0.0031	≈52	≈4.2
PlantAIMChai et al. (2025)	0.9692 ± 0.0037	0.9634 ± 0.0045	0.9588 ± 0.0052	0.9915 ± 0.0022	≈156	≈8.3
DARNDey et al. (2025)	0.9527 ± 0.0052	0.9445 ± 0.0069	0.9402 ± 0.0067	0.9859 ± 0.0034	≈27	≈10.9
PLDC-NetRichter and Kim (2026)	0.9618 ± 0.0044	0.9556 ± 0.0051	0.9508 ± 0.0060	0.9892 ± 0.0026	≈66	≈2.8
DFN-PSANDai et al. (2024)	0.9726 ± 0.0032	0.9671 ± 0.0040	0.9623 ± 0.0048	0.9931 ± 0.0019	≈121	≈9.3
PlantXViTThakur et al. (2022)	0.9654 ± 0.0041	0.9582 ± 0.0049	0.9559 ± 0.0055	0.9906 ± 0.0024	≈11	≈3.6
PD2SE-NetLiang et al. (2019)	0.9365 ± 0.0069	0.9278 ± 0.0080	0.9211 ± 0.0087	0.9783 ± 0.0042	≈96	≈5.1
FloraSyntropy-netKhan et al. (2026)	0.9748 ± 0.0035	0.9692 ± 0.0038	0.9657 ± 0.0042	0.9940 ± 0.0018	≈143	≈14.1
**Ours**	**0.9891 ± 0.0016**	**0.9911 ± 0.0014**	**0.9761 ± 0.0026**	**0.9995 ± 0.0003**	**≈35**	**≈6.2**

Bold represents the optimal value.

**Table 3:** Comparison results of different models on the PlantVillage dataset.

Method	Accuracy	Precision	Recall	AUC	Parameters (M)	Inference time (ms)
ResNet50	0.9012 ± 0.0075	0.8926 ± 0.0082	0.8841 ± 0.0090	0.9728 ± 0.0046	≈25	≈15.2
Vision-Transformer	0.9285 ± 0.0064	0.9173 ± 0.0076	0.9128 ± 0.0081	0.9843 ± 0.0037	≈112	≈6.7
RIC-Net	0.9478 ± 0.0056	0.9391 ± 0.0065	0.9324 ± 0.0072	0.9906 ± 0.0028	≈74	≈12.5
APDC	0.9362 ± 0.0069	0.9255 ± 0.0073	0.9197 ± 0.0078	0.9874 ± 0.0031	≈52	≈4.2
PlantAIM	0.9569 ± 0.0049	0.9502 ± 0.0058	0.9445 ± 0.0063	0.9932 ± 0.0021	≈156	≈8.3
DARN	0.9427 ± 0.0060	0.9338 ± 0.0069	0.9271 ± 0.0074	0.9891 ± 0.0030	≈27	≈10.9
PLDC-Net	0.9514 ± 0.0052	0.9447 ± 0.0061	0.9383 ± 0.0067	0.9917 ± 0.0025	≈66	≈2.8
DFN-PSAN	0.9618 ± 0.0042	0.9560 ± 0.0050	0.9498 ± 0.0057	0.9944 ± 0.0018	≈121	≈9.3
PlantXViT	0.9536 ± 0.0051	0.9469 ± 0.0059	0.9416 ± 0.0061	0.9925 ± 0.0023	≈11	≈3.6
PD2SE-Net	0.9194 ± 0.0071	0.9081 ± 0.0084	0.9026 ± 0.0088	0.9801 ± 0.0040	≈96	≈5.1
FloraSyntropy-net	0.9643 ± 0.0039	0.9584 ± 0.0047	0.9526 ± 0.0054	0.9951 ± 0.0016	≈143	≈14.1
**Ours**	**0.9800 ± 0.0024**	**0.9758 ± 0.0030**	**0.9727 ± 0.0035**	**0.9989 ± 0.0004**	**≈35**	**≈6.2**

Bold represents the optimal value.

As shown in **Tables 2**, **3**, the algorithm proposed in this paper achieves the best overall performance on both the NGLD and PlantVillage datasets, indicating that MF-SSFE and the conformal-aware reliable prediction mechanism can effectively improve the discriminative ability and prediction stability of plant disease classification models. On the NGLD dataset, the algorithm proposed in this paper achieves Accuracy, Precision, Recall, and AUC values of 0.9891, 0.9911, 0.9761, and 0.9995, respectively, outperforming ResNet50, Vision Transformer, and various feature fusion and attention-enhanced models. This demonstrates that multifrequency decomposition can more fully extract low-frequency structural variations and high-frequency lesion textures in grape leaf diseases, while selective state-space modeling further enhances the relational representation among cross-region disease features. On the PlantVillage dataset, although the image backgrounds are relatively regular and the category annotations are clear, and some advanced models have already achieved high performance, the algorithm proposed in this paper still maintains the best results across all four metrics. This indicates that the proposed method is not only applicable to more targeted single-crop disease recognition scenarios, but also maintains stable advantages in multi-crop and multi-class controlled-environment datasets. Compared with feature fusion methods such as PlantAIM, DFN-PSAN, and FloraSyntropy-net, the advantage of the method proposed in this paper comes not only from feature enhancement itself, but also from the further constraint imposed by conformal-aware reliable prediction on the classification output distribution. As a result, the model improves the quality of disease evidence representation while reducing overconfident predictions, thereby obtaining more stable classification results. Furthermore, this paper also achieved good results on the difficult dataset PlantDoc, as shown in **Table 4**.

**Table 4:** Comparison results of different models on the PlantDoc dataset.

Method	Accuracy	Precision	Recall	AUC	Parameters (M)	Inference Time (ms)
ResNet50	0.2063 ± 0.0158	0.1817 ± 0.0189	0.1645 ± 0.0204	0.7428 ± 0.0127	≈25	≈15.2
Vision-Transformer	0.2684 ± 0.0136	0.2369 ± 0.0155	0.2241 ± 0.0163	0.8014 ± 0.0098	≈112	≈6.7
RIC-Net	0.3127 ± 0.0119	0.2842 ± 0.0136	0.2705 ± 0.0148	0.8321 ± 0.0089	≈74	≈12.5
APDC	0.2916 ± 0.0142	0.2635 ± 0.0167	0.2518 ± 0.0174	0.8183 ± 0.0104	≈52	≈4.2
PlantAIM	0.3369 ± 0.0107	0.3114 ± 0.0129	0.2976 ± 0.0135	0.8475 ± 0.0076	≈156	≈8.3
DARN	0.3048 ± 0.0128	0.2762 ± 0.0144	0.2609 ± 0.0151	0.8266 ± 0.0095	≈27	≈10.9
PLDC-Net	0.2815 ± 0.0153	0.2511 ± 0.0172	0.2397 ± 0.0180	0.8097 ± 0.0109	≈66	≈2.8
DFN-PSAN	0.3452 ± 0.0101	0.3198 ± 0.0120	0.3074 ± 0.0127	0.8536 ± 0.0072	≈121	≈9.3
PlantXViT	0.3261 ± 0.0114	0.2986 ± 0.0132	0.2869 ± 0.0141	0.8408 ± 0.0081	≈11	≈3.6
PD2SE-Net	0.2487 ± 0.0166	0.2184 ± 0.0191	0.2033 ± 0.0207	0.7832 ± 0.0116	≈96	≈5.1
FloraSyntropy-net	0.3524 ± 0.0098	0.3276 ± 0.0118	0.3162 ± 0.0123	0.8589 ± 0.0069	≈143	≈14.1
**Ours**	**0.3889 ± 0.0072**	**0.3570 ± 0.0085**	**0.3377 ± 0.0094**	**0.8690 ± 0.0058**	**≈35**	**≈6.2**

Bold represents the optimal value.

As shown in **Table 4**, the overall performance on the PlantDoc dataset is significantly lower than that on NGLD and PlantVillage, which is mainly related to its complex real-world backgrounds, diverse crop types, small inter-class visual differences, and imbalanced sample distribution. Nevertheless, the method proposed in this paper still achieves the best results on this challenging dataset, with Accuracy, Precision, Recall, and AUC reaching 0.3889, 0.3570, 0.3377, and 0.8690, respectively, all of which are higher than those of the comparison models. Compared with ResNet50 and Vision Transformer, the method proposed in this paper not only improves the overall classification accuracy, but also shows more stable category recognition ability in terms of Precision and Recall, indicating that relying solely on local convolutional features or global self-attention modeling is insufficient to fully handle the complex backgrounds and finegrained disease differences in PlantDoc. Compared with feature enhancement or fusion methods such as RIC-Net, PlantAIM, DFN-PSAN, and FloraSyntropy-net, the method proposed in this paper further uses the MF-SSFE module to jointly enhance low-frequency leaf structures, highfrequency lesion textures, and cross-region disease contexts, enabling the model to distinguish easily confused disease categories in natural scenes more effectively. Meanwhile, the higher AUC indicates that although the model is still limited by the complexity of the dataset in top-1 classification, its category ranking ability and overall discriminative trend are more stable. This also suggests that the conformal-aware reliable prediction mechanism can alleviate overconfident predictions on difficult samples and provide more reliable decision support for plant disease recognition in complex real-world scenarios.

### 4.5 Prediction-set performance evaluation

To evaluate the reliability and informativeness of the conformal prediction mechanism, prediction-set experiments are conducted on the NGLD, PlantDoc, and PlantVillage datasets under the significance level *α* = 0.10. The empirical marginal coverage rate, average prediction set size, and singleton prediction rate are adopted to assess whether the generated candidate sets simultaneously maintain sufficient coverage and compactness.

As shown in **Table 5**, the prediction-set behavior varies substantially across the three datasets. NGLD and PlantVillage achieve empirical coverage rates of 0.8968 and 0.9011, respectively, which are close to the nominal coverage level of 0.90 corresponding to *α* = 0.10. Meanwhile, their average prediction set sizes remain close to one, and their singleton rates exceed 0.94, indicating that most samples can be assigned compact and highly specific prediction sets. By contrast, PlantDoc exhibits a much lower Top-1 accuracy of 0.3889, while its empirical coverage still reaches 0.9078, which is also close to the nominal coverage level. Its larger average set size of 2.68 and lower singleton rate of 0.2864 indicate that the model more frequently retains multiple plausible disease categories for difficult natural-scene samples. These results suggest that the conformal prediction mechanism adapts the prediction set according to sample difficulty, producing compact outputs for relatively easy samples while preserving broader candidate sets when substantial classification uncertainty is present.

**Table 5:** Prediction-set results on the three datasets at *α* = 0.10.

Dataset	Top-1 accuracy	Empirical coverage	Average set size	Singleton rate
NGLD	0.9891	0.8968	1.03	0.9732
PlantDoc	0.3889	0.9078	2.68	0.2864
PlantVillage	0.9800	0.9011	1.06	0.9447

### 4.6 Ablation experimental results

To further verify the contribution of each core module to the overall model performance, this paper designs ablation experiments under the same experimental settings and progressively introduces the MF-SSFE module and the CARP module for comparative analysis. Through the comparison among different model variants, the roles of multi-frequency selective state-space feature enhancement and conformal-aware reliable prediction in plant disease classification tasks can be evaluated more clearly. The experimental results are shown in **Table 6**.

**Table 6:** Ablation experimental results and approximate computational overhead on three plant disease classification datasets.

Dataset	Method	Accuracy	Precision	Recall	AUC	Parameters (M)	Inference time (ms)
NGLD	Baseline	0.9738 ± 0.0034	0.9582 ± 0.0047	0.9517 ± 0.0051	0.9962 ± 0.0011	≈28	≈4.8
	+MF-SSFE	0.9826 ± 0.0021	0.9748 ± 0.0039	0.9685 ± 0.0028	0.9987 ± 0.0007	≈35	≈5.4
	+CARP	0.9799 ± 0.0029	0.9816 ± 0.0024	0.9631 ± 0.0042	0.9982 ± 0.0009	≈28	≈5.0
	**Ours**	**0.9891 ± 0.0016**	**0.9911 ± 0.0014**	**0.9761 ± 0.0026**	**0.9995 ± 0.0003**	**≈35**	**≈6.2**
PlantDoc	Baseline	0.3007 ± 0.0115	0.2520 ± 0.0132	0.2344 ± 0.0108	0.8336 ± 0.0091	≈28	≈4.8
	+MF-SSFE	0.3541 ± 0.0096	0.3153 ± 0.0118	0.2914 ± 0.0127	0.8519 ± 0.0064	≈35	≈5.4
	+CARP	0.3378 ± 0.0124	0.3296 ± 0.0097	0.3042 ± 0.0113	0.8587 ± 0.0076	≈28	≈5.0
	**Ours**	**0.3889 ± 0.0072**	**0.3570 ± 0.0085**	**0.3377 ± 0.0094**	**0.8690 ± 0.0058**	**≈35**	**≈6.2**
PlantVillage	Baseline	0.9486 ± 0.0063	0.9424 ± 0.0071	0.9357 ± 0.0068	0.9952 ± 0.0018	≈28	≈4.8
	+MF-SSFE	0.9662 ± 0.0045	0.9635 ± 0.0049	0.9589 ± 0.0057	0.9981 ± 0.0012	≈35	≈5.4
	+CARP	0.9615 ± 0.0058	0.9552 ± 0.0064	0.9638 ± 0.0042	0.9974 ± 0.0015	≈28	≈5.0
	**Ours**	**0.9800 ± 0.0024**	**0.9758 ± 0.0030**	**0.9727 ± 0.0035**	**0.9989 ± 0.0004**	**≈35**	**≈6.2**

Bold represents the optimal value.

**Figure 4:**
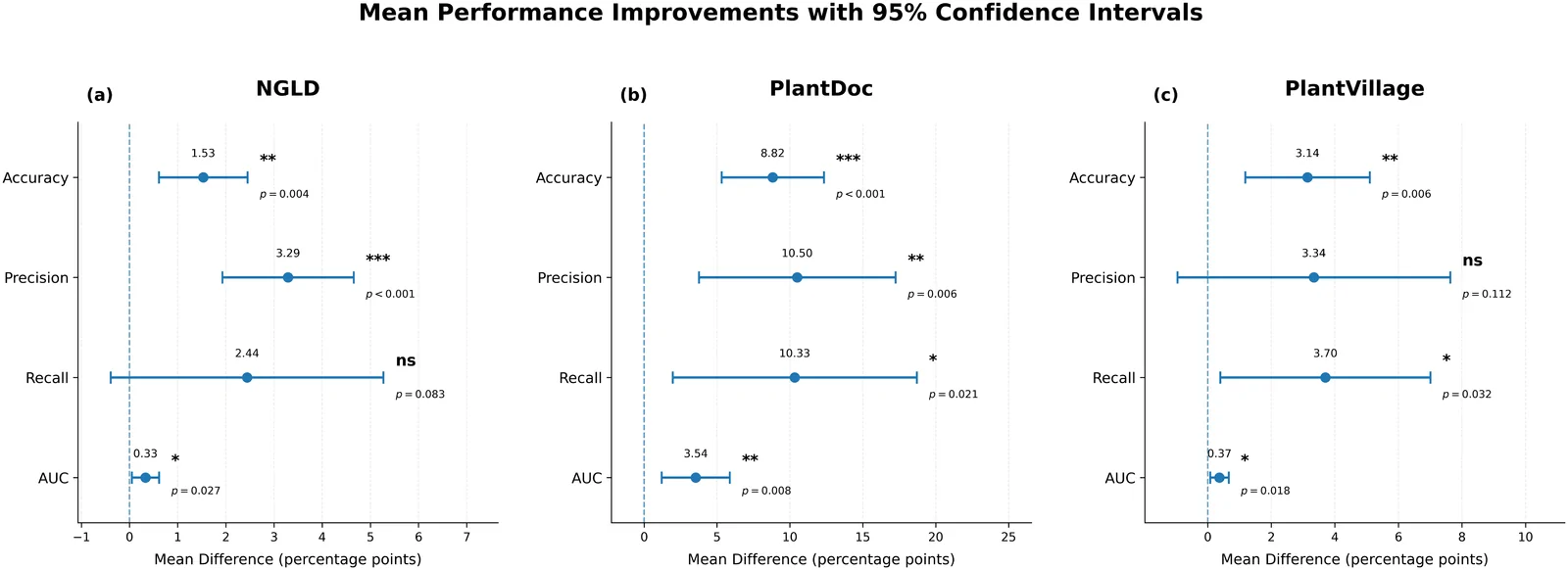
The mean performance differences and their 95% confidence intervals between the proposed method and the baseline models on the NGLD, PlantDoc, and PlantVillage datasets. Positive values indicate that the proposed method outperforms the corresponding baseline model. Statistical significance was determined using a two-sided paired *t*-test, where *** denotes *p <* 0.001, ** denotes *p <* 0.01, * denotes *p <* 0.05, and ns denotes *p* ≥ 0.05.

As shown in **Table 6**, both the MF-SSFE and CARP modules bring stable performance gains across different datasets, indicating that the improvement of the proposed framework does not come from a single structural modification, but from the joint effect of multi-frequency disease feature enhancement and reliable prediction constraints. Compared with the Baseline, after introducing MF-SSFE, Accuracy, Precision, Recall, and AUC are improved on all three datasets, demonstrating that low-frequency leaf structure, high-frequency lesion texture, and selective state-space contextual modeling can effectively enhance the discriminative ability of the model for disease-related regions. After introducing CARP alone, the model also shows obvious improvements in Precision, Recall, and AUC, especially on complex real-world datasets such as PlantDoc, where prediction reliability is further improved. This indicates that the conformal-aware constraint helps alleviate the overconfidence problem of the model on visually similar categories and difficult samples. The complete model, Ours, achieves the best results across all three datasets and four evaluation metrics, further proving the good complementarity between MF-SSFE and CARP: the former improves the quality of visual features, while the latter optimizes the reliability and stability of the prediction distribution, thereby jointly enhancing the overall performance of plant disease classification models in both controlled scenarios and complex natural scenes. Finally, we also present a significance analysis, as shown in **Figure 4**.

As shown in **Figure 4**, the mean performance differences achieved by the proposed method are generally positive across the three datasets, although the statistical significance varies among different evaluation metrics. The improvement in Accuracy on PlantDoc reaches the highly significant level, while the AUC improvements on all three datasets are statistically significant, indicating that the corresponding performance gains remain relatively consistent across repeated experiments. In contrast, the improvement in Recall on NGLD and that in Precision on PlantVillage do not reach statistical significance, suggesting that the observed gains in these two metrics may still be affected by experimental variability. Therefore, these results should be interpreted cautiously by jointly considering the mean performance differences and their corresponding confidence intervals.

### 4.7 Visualize experimental results

#### 4.7.1 t-SNE experimental results

**Figure 5:**
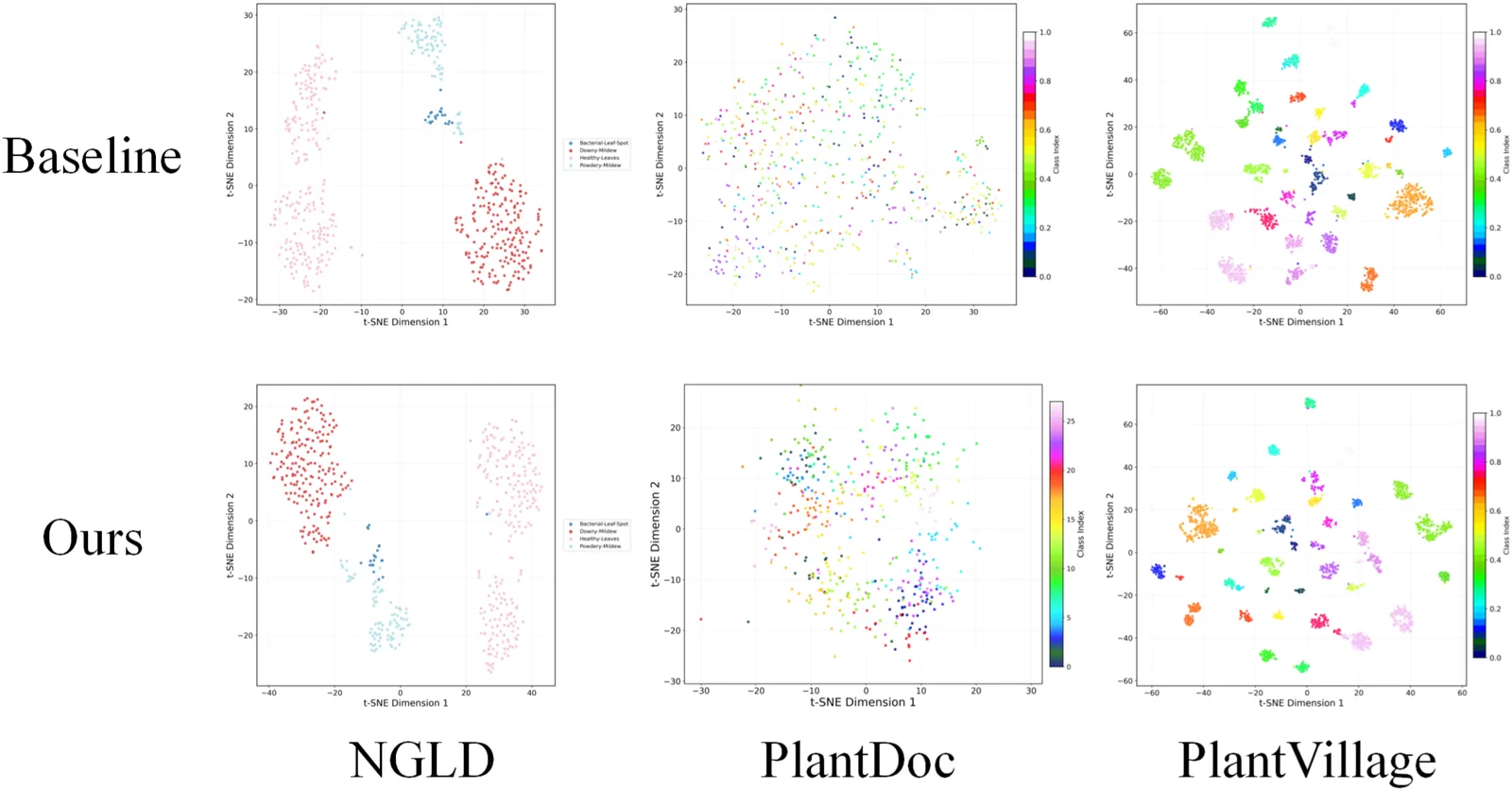
t-SNE visualization experimental results.

To further analyze the category distribution of different models in the feature space, this paper adopts t-SNE to visualize the high-dimensional feature representations of test samples in a two-dimensional space, and the experimental results are shown in **Figure 5**. This experiment aims to intuitively show whether the disease features learned by the model have good intra-class compactness and inter-class separability, thereby further supporting the representation ability of the proposed method in complex plant disease classification tasks. By comparing the feature distributions of Baseline and Ours, the influence of multi-frequency selective state-space feature enhancement and conformal-aware reliable prediction on discriminative feature learning can be observed more intuitively.

As shown in the figure, the t-SNE visualization results on different datasets are basically consistent with the trends observed in the quantitative experiments. For the NGLD dataset, the Baseline model can already form a certain degree of category separation, but sample overlap still exists in some local regions. In contrast, Ours presents a more compact intra-class sample distribution and clearer category boundaries, indicating that the proposed modules can further enhance discriminative representations in single-crop disease scenarios. For the PlantDoc dataset, due to its complex backgrounds, large number of categories, and small inter-class differences, the feature distribution of the Baseline model shows an obvious mixed pattern and it is difficult to form stable clusters. By comparison, although Ours still has a certain degree of overlap, its overall distribution shows a clearer local clustering trend, indicating that the joint modeling of high-frequency lesion textures, low-frequency leaf structures, and long-range contextual relationships by MF-SSFE helps improve feature separability in complex scenarios. For the PlantVillage dataset, Ours shows more obvious intra-class compactness and inter-class dispersion than the Baseline, further demonstrating that the conformal-aware reliable prediction mechanism can constrain the model output distribution on the basis of feature representation, making the learned disease representations more stable.

#### 4.7.2 AUROC experimental results

**Figure 6:**
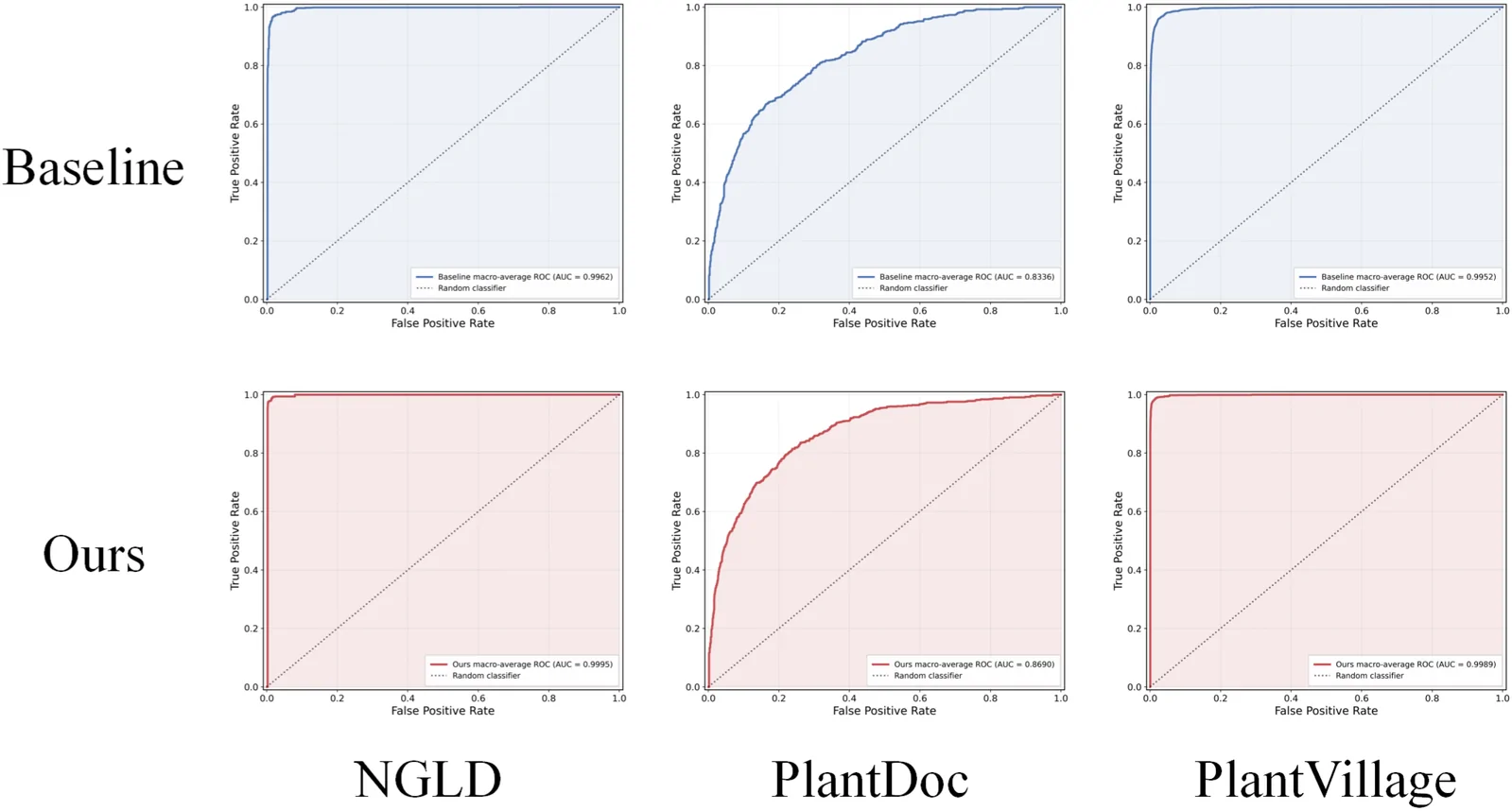
AUROC visualization experimental results.

To further evaluate the overall discriminative ability of the model under different classification thresholds, this paper plots AUROC curves to visually analyze the classification ranking performance of Baseline and Ours. Different from Accuracy, Precision, and Recall under a single threshold, AUROC can reflect the ability of the model to distinguish positive and negative samples as well as multi-class samples from a more comprehensive perspective. Therefore, it is suitable for analyzing the stability and reliability of plant disease classification models on different datasets. By comparing the AUROC curves of different methods, the performance of the proposed method in terms of probability output quality and class-discriminative boundaries can be further observed. The experimental results are shown in **Figure 6**.

As shown in the figure, the AUROC visualization results further verify the classification discriminative ability of the proposed method on different datasets. For the NGLD and PlantVillage datasets, the curve of the Baseline is already close to the upper-left corner, indicating that the category distinctions in these two datasets are relatively clear. However, the curve of Ours is overall closer to the boundary of an ideal classifier, demonstrating that the proposed method can further optimize the quality of probability ranking on the basis of high performance. For the more challenging PlantDoc dataset, there is still a noticeable gap between the Baseline curve and the ideal upper-left corner, reflecting that complex backgrounds, multi-class differences, and similar disease patterns have a greater impact on model discrimination. In contrast, the curve of Ours rises faster in the low false positive rate region and maintains a higher true positive rate across the overall range, indicating that the enhancement of disease-related features by MF-SSFE and the reliability constraint imposed by CARP can improve the ranking stability and category discrimination ability of the model on difficult samples. Overall, the AUROC results show that the proposed method not only improves classification performance under a single threshold, but also enhances the comprehensive discriminative performance of the model under different threshold conditions.

### 4.8 Sensitivity analysis of the conformal significance level

To further analyze the influence of the significance level *α* in the conformal-aware reliable prediction module on model classification performance and reliability constraints, this paper conducts sensitivity experiments with different *α* values on three plant disease classification datasets. The significance level *α* directly affects the construction threshold of candidate prediction sets in conformal prediction, and further influences the balance among class discrimination, probability ranking, and uncertainty expression. Therefore, by comparing Accuracy, Precision, Recall, and AUC under different *α* settings, the stability of the proposed method with respect to this key hyperparameter can be evaluated more comprehensively, providing a basis for the selection of *α* in subsequent experiments. The experimental results are shown in **Table 7**.

**Table 7:** Sensitivity analysis results under different significance levels *α*.

Dataset	α	Accuracy	Precision	Recall	AUC	Empirical coverage	Average set size
NGLD	0.05	0.9868	0.9879	0.9740	0.9992	0.9445	1.08
	0.10	0.9891	0.9911	0.9761	0.9995	0.8968	1.03
	0.15	0.9876	0.9885	0.9748	0.9993	0.8437	1.03
	0.20	0.9859	0.9864	0.9725	0.9988	0.7992	1.01
	0.25	0.9837	0.9849	0.9702	0.9983	0.7477	1.00
PlantDoc	0.05	0.3812	0.3465	0.3284	0.8642	0.9546	3.10
	0.10	0.3889	0.3570	0.3377	0.8690	0.9078	2.68
	0.15	0.3835	0.3516	0.3321	0.8664	0.8575	2.32
	0.20	0.3759	0.3438	0.3250	0.8617	0.7961	2.02
	0.25	0.3672	0.3364	0.3178	0.8569	0.7450	1.79
PlantVillage	0.05	0.9764	0.9712	0.9686	0.9985	0.9476	1.14
	0.10	0.9800	0.9758	0.9727	0.9989	0.9011	1.06
	0.15	0.9778	0.9736	0.9709	0.9986	0.8499	1.02
	0.20	0.9751	0.9704	0.9675	0.9981	0.7945	1.01
	0.25	0.9718	0.9669	0.9634	0.9975	0.7523	1.00

**Figure 7:**
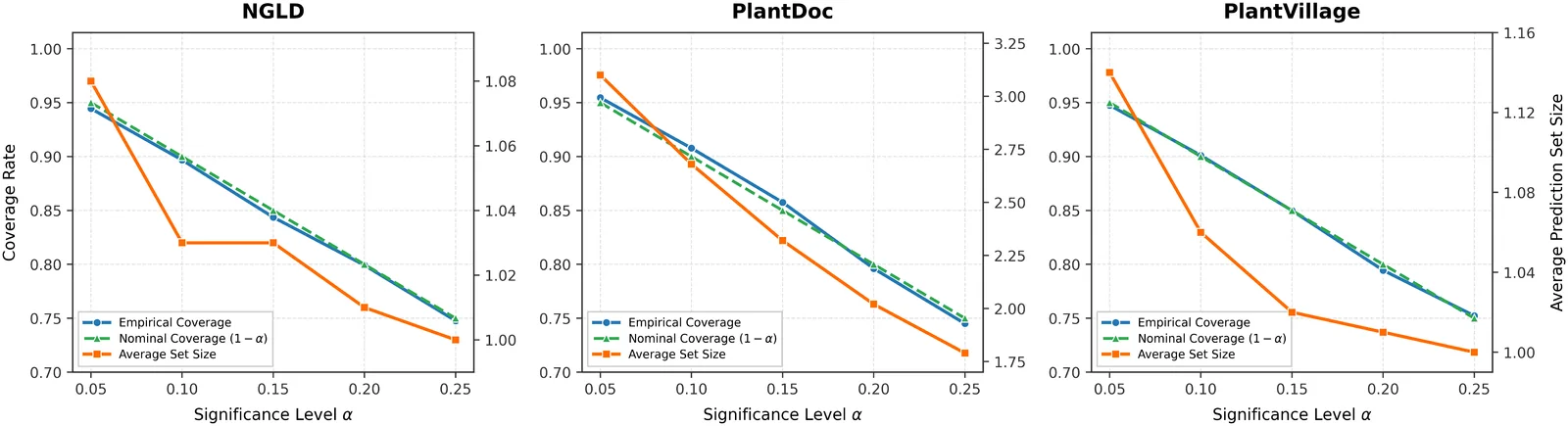
Empirical coverage, nominal coverage, and average prediction set size under different significance levels *α* on the NGLD, PlantDoc, and PlantVillage datasets.

As shown in **Table 7**, the significance level *α* has a certain influence on model performance, but the proposed method maintains good overall stability under different values. When *α* = 0.10, the model achieves the best results on the NGLD, PlantDoc, and PlantVillage datasets, indicating that this setting can form a more reasonable balance between reliability constraints and classification discriminability. When *α* is smaller, conformal prediction imposes stricter coverage requirements on the prediction set, and the model tends to retain more candidate classes. Although this helps reduce the risk of missed recognition, it may weaken the compactness of the classification boundary, resulting in some metrics being slightly lower than those obtained when *α* = 0.10. As *α* continues to increase, the candidate set constraint is gradually relaxed, and the model output becomes more aggressive. Although the prediction set may become more compact, the tolerance for uncertain samples decreases, leading to varying degrees of decline in Accuracy, Precision, Recall, and AUC. Especially on complex natural-scene datasets such as PlantDoc, the performance fluctuations caused by different *α* settings are more obvious, indicating that complex backgrounds and fine-grained category differences are more sensitive to reliability constraints. Overall, *α* = 0.10 achieves relatively better classification performance and stable probability ranking ability on all three datasets; therefore, this setting is adopted as the default significance level in the subsequent experiments. Finally, this paper also presents a graph showing how empirical coverage and average prediction set size change with *α*, as shown in **Figure 7**.

As shown in **Figure 7**, increasing *α* consistently reduces both the empirical coverage rate and the average prediction set size across the three datasets, reflecting the expected trade-off between prediction reliability and specificity. The empirical coverage generally remains close to the corresponding nominal coverage level of 1 − *α*, indicating that the conformal prediction mechanism provides appropriately calibrated prediction sets under different significance levels. PlantDoc maintains substantially larger prediction sets than NGLD and PlantVillage because its complex natural backgrounds, greater inter-class similarity, and lower Top-1 accuracy require the model to retain more candidate categories to achieve the desired coverage. In contrast, the prediction sets on NGLD and PlantVillage remain close to singleton sets across different significance levels, reflecting the stronger discriminative ability of the model on these two datasets. Overall, smaller *α* values produce broader prediction sets and higher empirical coverage, whereas larger *α* values generate more compact prediction sets at the cost of reduced coverage.

## 5 Limitations and discussion

Although the proposed method achieves the best comparative performance on the PlantDoc dataset under the same experimental protocol, its Top-1 accuracy of 38.89% remains low in absolute terms and is insufficient to support direct practical deployment. This result indicates that plant disease classification in unconstrained real-world environments remains highly challenging, particularly when the visual characteristics of disease categories are affected by complex natural backgrounds, substantial illumination variation, partial leaf occlusion, limited image resolution, and large appearance differences among crop varieties. Therefore, the PlantDoc results should be interpreted as evidence of a relative performance improvement under a difficult natural-scene setting rather than as proof of a deployment-ready diagnostic system. Although the conformal prediction mechanism provides candidate class sets and explicitly represents predictive uncertainty, it cannot fully compensate for severely insufficient diseaserelated evidence, substantial inter-class similarity, or distribution differences between controlled and real-world images.

**Figure 8:**
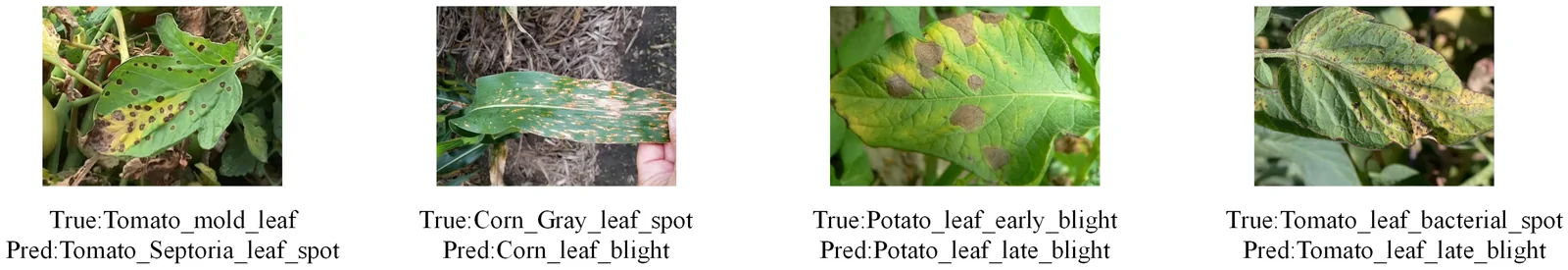
PlantDoc dataset failure example.

A qualitative examination of the incorrectly classified PlantDoc samples further reveals several recurring failure patterns. Images containing strong background clutter or irrelevant objects may produce responses similar to lesion boundaries and redirect model attention away from the actual diseased regions. Partial occlusion and small visible lesion areas reduce the amount of discriminative evidence available for classification, while low illumination, blur, noise, and compression artifacts weaken disease-related color and texture cues. In addition, considerable differences in leaf morphology and color among crop varieties increase intra-class variability, whereas visually similar symptoms across different crop–disease combinations lead to ambiguous inter-class boundaries. These failure cases indicate that the current framework remains sensitive to image quality degradation, incomplete lesion visibility, background interference, and cross-crop symptom similarity, which jointly limit its recognition reliability in complex field conditions. An example of its failure is shown in **Figure 8**.

A direct cross-dataset distribution-shift experiment between PlantVillage and PlantDoc was not conducted because the two datasets do not share a fully aligned label space and contain different crop species and crop–disease combinations. Under such conditions, directly training on PlantVillage and testing on PlantDoc would introduce not only image-domain variation but also substantial label-space and category-semantic mismatch, making it difficult to determine whether performance degradation is caused by background distribution shift, unseen crop categories, or unavailable disease labels. Moreover, empirical conformal coverage cannot be meaningfully interpreted when the ground-truth test category is absent from the training and calibration label space. Consequently, the absence of an evaluation under a strictly label-aligned distribution shift remains a limitation of the current study, and the reliability conclusions of CARP are restricted to the within-dataset experimental settings considered in this work. This study also provides experimental results using different backbones; further details are provided in the **Appendix**.

## 6 Conclusion

This paper proposes a reliable visual recognition framework for plant disease image classification. By introducing a multi-frequency selective state-space feature enhancement module and a conformal-aware reliable prediction module after the Swin-Tiny backbone network, the proposed framework achieves joint modeling of disease-discriminative feature enhancement and prediction reliability constraints. The MF-SSFE module enhances disease-related evidence from three aspects: low-frequency leaf structures, high-frequency lesion textures, and cross-region statespace contexts, enabling the model to more fully capture fine-grained disease differences under complex backgrounds. The CARP module further introduces the idea of conformal prediction into the model output process, so that the classification results include not only the conventional top-1 disease label, but also a candidate prediction set with uncertainty expression capability. Experimental results show that the proposed method achieves superior performance on the NGLD, PlantDoc, and PlantVillage datasets, and demonstrates good discriminative ability, stability, and balance between performance and computational overhead in ablation experiments, significance-level sensitivity analysis, t-SNE visualization, AUROC analysis, and model complexity analysis.

Future research can be further carried out from three aspects. First, the current method is mainly validated on image-level plant disease classification tasks. In future work, it can be further extended to more fine-grained tasks such as lesion localization, disease severity estimation, and multi-label disease recognition, so as to enhance its applicability in practical agricultural diagnosis scenarios. Second, although conformal-aware reliable prediction can improve the reliability of model outputs, data distribution shifts under cross-region, cross-crop, and cross-device acquisition conditions may still affect the coverage stability of prediction sets. Therefore, future work can combine domain generalization, test-time adaptation, and more robust calibration mechanisms to improve the generalization ability of the model in real field environments. Finally, considering the efficiency requirements of agricultural edge devices and mobile deployment, future research can further explore lightweight state-space modeling, knowledge distillation, and structural pruning strategies to reduce inference latency and deployment cost while maintaining reliable recognition capability.

## Data Availability

The original contributions presented in the study are included in the article/supplementary material. Further inquiries can be directed to the corresponding author.

## Appendix

**Table:** Table 8 Different backbone experimental results.

Backbone	NGLD accuracy	PlantDoc accuracy	PlantVillage accuracy	Parameters (M)	Inference time (ms)
ResNet50+Ours	0.9371 ± 0.0143	0.2276 ± 0.0104	0.9168 ± 0.0089	≈31	≈17.1
ConvNeXt-Tiny+Ours	0.9860 ± 0.0022	0.3805 ± 0.0087	0.9769 ± 0.0031	≈38	≈6.8
ViT-S/16+Ours	0.9851 ± 0.0025	0.3746 ± 0.0091	0.9752 ± 0.0034	≈126	≈8.9
**Swin-Tiny+Ours**	**0.9891 ± 0.0016**	**0.3889 ± 0.0072**	**0.9800 ± 0.0024**	**≈35**	**≈6.2**
Swin-Small+Ours	0.9894 ± 0.0017	0.3902 ± 0.0070	0.9806 ± 0.0023	≈57	≈8.9
Swin-Base+Ours	0.9900 ± 0.0015	0.3915 ± 0.0068	0.9812 ± 0.0021	≈94	≈12.7

Bold represents the optimal value.
